# Should we perform a FAST exam in haemodynamically stable patients presenting after blunt abdominal injury: a retrospective cohort study

**DOI:** 10.1186/s13049-016-0342-0

**Published:** 2017-01-03

**Authors:** D. Dammers, M. El Moumni, I.I. Hoogland, N. Veeger, E. ter Avest

**Affiliations:** 1 Department of Emergency Medicine, Medical Center Leeuwarden, Henry Dunantweg 2, 8934 AD Leeuwarden, The Netherlands; 2 Department of Trauma surgery, University of Groningen, University Medical Center Groningen, Groningen, The Netherlands; 3 Medical Student, University of Groningen, Groningen, The Netherlands; 4 Department of Epidemiology, University of Groningen, University Medical Center Groningen and Medical Center Leeuwarden, Groningen, The Netherlands

**Keywords:** Focussed Assessment with Sonography for Trauma, FAST, Blunt abdominal injury

## Abstract

**Background:**

Focussed Assessment with Sonography for Trauma (FAST) is a bedside ultrasonography technique used to detect free intraperitoneal fluid in patients presenting with blunt abdominal trauma (BAT) in the emergency department.

**Methods:**

In this retrospective cohort study we investigated the potential of FAST as a risk stratification instrument in haemodynamically (HD) stable patients presenting after BAT by establishing the association between the FAST exam result and final outcome. An adverse outcome was defined in this context as the need for either a laparoscopy/laparotomy *or* an angiographic embolization *or* death due to abdominal injuries).

**Results:**

A total of 421 patients with BAT were included, of which nine had an adverse outcome (2%). FAST was negative in 407 patients. Six of them turned out to have free intraperitoneal fluid (sensitivity 67 [41–86]%). FAST was positive in 14 patients, 12 of whom had free intraperitoneal fluid (specificity 99 [98–100]%). A positive FAST (positive likelihood ratio 34.3 [15.1–78.5]) was stronger associated with an adverse outcome than Injury Severity Score (ISS) or any individual clinical- or biochemical variables measured at presentation in the ED.

**Discussion:**

The FAST exam can provide valuable prognostic information at minimal expenses during the early stages of resuscitation in haemodynamically stable patients presenting with BAT.

**Conclusions:**

FAST exam should not be omitted in patients with BAT.

**Electronic supplementary material:**

The online version of this article (doi:10.1186/s13049-016-0342-0) contains supplementary material, which is available to authorized users.

## Background

Focussed Assessment with Sonography for Trauma (FAST) is a bedside ultrasonography technique used to detect free intraperitoneal fluid in patients presenting with blunt abdominal trauma (BAT) in the emergency department [1–7]. The FAST exam can be carried out quickly and reliably (both by radiologists and emergency physicians [8–13], at limited costs and without radiation exposure to the patient. Performing a FAST exam expedites time to definitive care [14–16], and thereby contributes to a better outcome for trauma patients. As a result, the use of FAST has been advocated by many guidelines and societies [17, 18], and FAST has become an integral part in the trauma-evaluation of patients with BAT.

Although the clinical benefit of early detection of free intra-abdominal fluid has been demonstrated in haemodynamically unstable patients with BAT, the advantage of performing a FAST exam in haemodynamically (HD) stable patients is less unequivocal. Previous studies have reported a relatively low sensitivity of FAST for the detection of free intraperitoneal fluid in these patients [6, 19–23]. Although the specificity of FAST for the detection of free intraperitoneal fluid is higher, computed tomographic (CT) confirmation is often preferred to decide on treatment (operative versus non-operative) when the FAST is positive [19]. Based on these findings, there is a tendency to discourage performing FAST in HD stable patients presenting after BAT.

Previous studies have primarily focussed on the diagnostic accuracy of FAST, and not on the qualities of FAST as a risk stratification tool. Therefore, in the present study, we aimed to investigate the value of FAST as an early risk stratification instrument in HD stable patients presenting after BAT.

## Methods

### Study design and setting

We performed a retrospective observational cohort study of all adult HD-stable patients who presented in the ED of a level 1 trauma center (University Medical Center Groningen) between June 1st 2014 and September 1st 2015 after BAT.

### Selection of participants

**Fig. 1:**
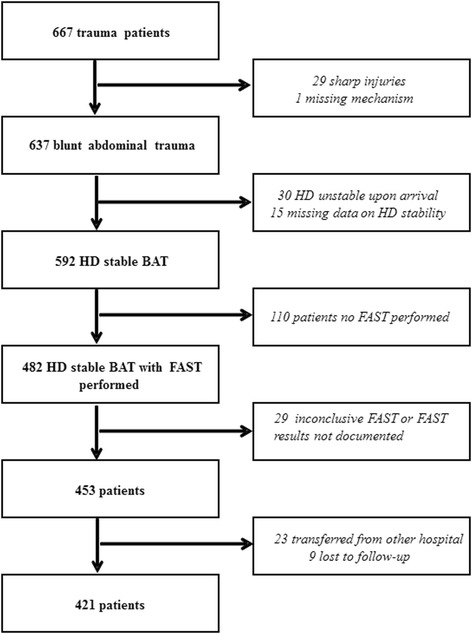
Flow chart of patient inclusion

Patients were selected from a prospectively kept trauma registry of the department of Trauma Surgery. Patients were included in the present study when they were > 18 years and presented with BAT. Inclusion was irrespective of trauma mechanism (fall from height, motor vehicle collision, etc.) or trauma severity (ISS score at discharge). Only HD stable patients were included. Haemodynamic status was defined based on the first available set of vital signs after presentation in the hospital. A systolic blood pressure cut-off value of >90 mmHg was used to differentiate HD-stable- from unstable patients. Patients were excluded when no FAST was performed during primary assessment, when FAST results were inconclusive (no clear visualisation of all three pouches), or when follow-up data regarding clinical outcome were unavailable (Fig. 1).

Data abstraction from the trauma registry was performed by two investigators (DD and IH). When information in the trauma registry was incomplete, the electronic hospital records were searched to identify missing information.

### FAST

FAST exam is an integral part of the trauma evaluation in the ED of the University Medical Center Groningen. All FAST-exams are performed by radiologists or radiology residents supervised by radiologists, using a Zonare ZS3 Premium Ultrasound System (Zonare Medical Systems, Inc. Mountain View, California, USA) with a C6-2 curved array transducer according to a standardized protocol, in which three pouches (hepatorenal, splenorenal and rectovesicular) are studied for the presence or absence of free intraperitoneal fluid. The FAST examination result is documented either as positive or negative. The FAST exam is considered positive when free intra-abdominal fluid was visualized in one of the three aforementioned pouches, and negative when it is absent in all three pouches.

### Outcome definitions

A true positive FAST was defined as the presence of free intraperitoneal fluid confirmed by CT or laparoscopy/laparotomy. A false negative FAST was defined as a negative FAST with confirmed free intraperitoneal fluid on CT. A true negative FAST was defined as a negative FAST in the absence of intraperitoneal fluid on a subsequently performed CT *or* a negative FAST in the absence of signs of abdominal bleeding on clinical follow up (no recorded haemodynamic instability and no recorded interventions like blood transfusions, angiographic embolization, or laparoscopy/laparotomy being performed). An *adverse outcome* was defined as the presence of an abdominal injury requiring either a critical intervention (either a laparoscopy/laparotomy or an angiographic embolization) or resulting in death during hospitalisation following ED presentation.

### Analysis

Data are represented as mean (95% CI) unless stated otherwise. Differences between FAST positive- and negative groups were tested by Mann-Whitney *U*-test or Fisher’s exact test where appropriate. Univariate logistic regression analysis was carried out to evaluate the association of various clinical- and biochemical variables (including the FAST exam) at presentation with outcome. Optimal cut-off values to discriminate between subjects with- and without an *adverse outcome* were determined for all continuous variables with an r >0.2 using ROC statistics under the condition of equal “cost” of misclassification of cases and non-cases. Likelihood ratio’s, sensitivities and specificities were calculated for these optimal cut off values in order to be able to compare the risk stratifying abilities of FAST with clinical- and biochemical variables. Base excess was chosen over HCO3- and pH as representative of the pertinent parameter (acid-base status). Missing data are reported in the results section according to the STARD 2015 guideline [24]. A *p*-value <0.05 was regarded as statistically significant. All statistical analyses were done using SPSS 23.0 for Windows statistical package (SPSS Inc., Chicago Illinois, USA).

As our study only involved retrospective evaluation of routinely recorded patient data, this type of study was determined to be exempt research by the ethical review board of the UMCG (METC UMCG, reference number 2016/007).

## Results

### Characteristics of study population

During the study period 667 trauma patients visited the ED. 637 of them presented with BAT, of which 216 did not meet our inclusion criteria: 30 patients were HD-unstable upon arrival in the ED, and for another 15 patients SBP on arrival was unavailable, and therefore HD-stability could not be established. In 110 patients no FAST exam was performed, in 10 patients a FAST was performed, but results were inconclusive, and in 19 patients FAST results were unavailable. 23 patients were transferred from another hospital to our ED. For 9 patients follow-up data regarding their clinical outcome were unavailable. Further results refer to the remaining group of 421 patients (Fig. 1).

Table 1 shows the patient characteristics of our study population stratified by FAST result. Patients with a positive FAST tended to be younger and were more often involved in motor vehicle collisions compared to patients with a negative FAST. At presentation, they had a higher respiratory rate and a lower GCS, whereas other vitals were not significantly different. Patients with a positive FAST had a lower haemoglobin (Hb) level, higher AST and ALT levels, a higher Creatinin Kinase (CK) and a higher leucocyte count (WBC). Their ISS score was higher, and critical interventions to stabilize their vital signs in the prehospital environment or in the ED were performed more often in this group. Subgroup analysis of the 110 patients in whom no FAST exam was performed revealed that none of the vital signs or biochemical results was significantly different from those in patients with a true negative FAST result.

**Table 1:** Patient characteristics of haemodynamically stable patients presenting after blunt abdominal injury stratified by FAST-exam result

	Positive FAST (*n* = 14)	Negative FAST (*n* = 401)	Missing
Demographics			
Age mean	35 (24–45)*	44 (42–46)	
Gender (men) n (%)	10 (71%)	265 (65%)	
Medication n (%)			
Vitamin K antagonist or LMWH	0	47 (12%)	8
Trauma mechanism n (%)			
MVC	7 (50%)	131 (32%)	
Motorcycle	2 (14%)	30 (7%)	
Scooter/moped	0	18 (4%)	
Bicycle	1 (7.1%)	59 (14%)	
Pedestrian	1 (7.1%)	12 (3%)	
Beaten with blunt object	0	6 (2%)	
Low-energy fall	0	63 (16%)	
High-energy fall^a^	2 (14%)	50 (13%)	
Other blunt trauma	1 (7.1%)	37 (9%)	
Vital Signs			
HR (bpm)	91 (77–104)	81 (79–84)	17
SBP (mm Hg)	127 (113–140)	140 (136–144)	
DBP (mm Hg)	84 (68–99)	84 (81–87)	1
RR (rpm)	27 (13–37)*	18 (17–19)	98
Saturation (%)	97 (92–101)	97 (95–99)	9
Temperature (°C)	35.6 (34.8–36.5)	36.1 (35.6–36.6)	208
GCS score (range)	12 (3–15)**	14 (3–15)	73
Laboratory values			
Hb (mmol/L)	7.3 (6.3–8.3)**	8.7 (8.3–9.0)	6
Leucocytes (x10^9/L)	17.7 (13.7–21.8)**	12.9 (11.4–14.5)	9
Thrombocytes (x10^9/L)	223 (184–261)	243 (236–249)	8
Bilirubin total (μmol/L)	6 (3–8)	7 (6–7)	12
Amylase (U/L)	61 (53–68)	66 (55–77)	12
AST (U/L)	310 (76–544)**	54 (46–63)	9
CK tot (U/L) (range)	566 (142–1214)**	445 (300–588)	23
Creatinine (μmol/L)	84 (77–90)*	74 (71–78)	26
Lactate (mmol/L)	2.9 (1.6–4.2)	2.2 (1.9–2.5)	178
BE (mmol/l)	–6 (−11 – −1)	−2 (−2 – −1)	223
HCO3- (mmol/l)	20 (17–23)	23 (22–23)	223
pH	7.25 (7.13–7.38)	7.38 (7.37–7.39)	223
PT (sec)	12.8 (11.6–14.0)**	12 (11–13)	36
aPTT (sec)	33 (17–49)	25 (25–26)	50
Interventions and Injury severity score			
Pre-hospital or ED Intubation	10 (71%)**	84 (21%)	
Pre-hospital or ED CPR	3 (21.1%)**	4 (0.9%)	
Pre-hospital or ED thoracostomy	4 (28%)**	30 (7%)	
ISS score	44 (37–52)**	16 (14–17)	

*LMWH* low molecular weight heparin, *MVC* motor vehicle collision, *SBP* systolic blood pressure, *HR* heart rate, DBP, diastolic blood pressure, *RR* respiratory rate, *GCS* Glasgow Coma Scale, *Hb* hemoglobin, *CK* creatine kinase, *PT* prothrombin time, *aPTT* activated partial thromboplastin time, *ISS* Injury Severity Score, *ED* emergency department, *CPR* cardiopulmonary resuscitation, *RBC* red blood cells, *FFP* fresh frozen plasma

*denotes *p* < 0.05 “compared to negative FAST”; **denotes *p* < 0.01 compared to “negative FAST”

^a^High-energy fall: from height >2-3x body length

### Accuracy of FAST

FAST was negative in 407 patients. Six of these had free intraperitoneal fluid (sensitivity 67 [41–86]%). FAST was positive in 14 patients, of which 12 had free intraperitoneal fluid (specificity 99 [98–100]%). Most of the patients with a positive FAST were significantly injured, as reflected by their mean ISS score of 44 (range 27–70) FAST results were confirmed by laparotomy (*n* = 2), CT-scanning (*n* = 69) or observation (*n* = 352). Underlying injuries found on CT in patients with positive- and false negative FAST-exams are presented in Table 2.

**Table 2:** CT-findings in haemodynamically stable blunt trauma patients with either a (true- or false) positive FAST (*n* = 14) or a false negative FAST (*n* = 6)

Subject nr	Free abdominal fluid confirmed (yes/no)	Findings on CT
Positive FAST		
1	Yes	Spleen laceration grade 4, active bleeding Liver laceration grade 2, no active bleeding
2	Yes	Deep laceration of liver into vena cava inferior. No active bleeding
3	Yes	No source identification for free fluid
4	Yes	Active bleeding of the mesentery
5	Yes	Spleen laceration grade 2, no active bleeding
6	Yes	Image of liver laceration grade 5/6, no active bleeding
7	Yes	Bruised liver, no active bleeding
8	Yes	Spleen laceration, no active bleeding
9	Yes	No source identification for free fluid
10	Yes	Liver laceration grade 5, no active bleeding Spleen laceration grade 5, no active bleeding
11	Yes	Spleen laceration, active bleeding
12	Yes	Liver laceration, suggestive for active bleeding Diffuse laceration of the spleen, active bleeding
13	No	No pathologic findings identified
14	No	No pathologic findings identified
False negative FAST		
15	Yes	No source identification for free fluid
16	Yes	Liver laceration grade 7/8, no active bleeding
17	Yes	Liver laceration grade 4
18	Yes	Free gas in the omental bursa, cave perforation.
19	Yes	Possible liver laceration, no active bleeding
20	Yes	Kidney and liver laceration, active bleeding

### In-hospital treatment and outcome of patients with BAT

An *adverse outcome* was encountered in a total of 9 patients (2%). Patients with a positive- or false-negative FAST exam more often had an adverse outcome (*n* = 8 vs *n* = 1, *p* < 0.01), and more often received blood transfusions than patients with a true negative FAST (*p* < 0.01). In addition, they were hospitalised longer: Mean duration of hospitalisation was 16.4 days (*p* < 0.01) for patients with a positive FAST, 9.2 days for patients with a false negative FAST and 6.6 days for patients with a true negative FAST (Table 3).

**Table 3:** Treatment of haemodynamically stable patients presenting after blunt abdominal injury stratified by FAST-exam result

	Positive FAST (*n* = 14)	False negative FAST (*n* = 6)	True negative FAST (*n* = 401)
Intervention n(%)			
Embolization	3 (21%)**	1 (17%)*	1 (0.2%)
Laparotomy	2 (14%) **	1 (17%)*	0
Transfusion during ED-stay			
For all indications	5 (36%)**	3 (50%)*	18 (4.5%)
For (presumed) abdominal bleeding	3 (21%)**	1 (17%)*	0
Transfusion during hospitalisation			
For all indications	4 (28%)**	1 (17%)*	22 (5.5%)
For abdominal bleeding	2 (14%)**	0	0
Destination after ED n(%)			
Discharged	0*	0	115 (29%)
Surgery ward	0**	2 (33%)	185 (46%)
ICU	13 (92.9%)**	4 (67%)*	100 (25%)
Deceased at ED	1 (7.1%)	0	1 (0.2%)
Duration of hospitalisation			
Hospitalisation days (range)	16.4 (0–31)**	9.2 (1–30)	6.6 (0–61)
Duration ICU days (range)	7.0 (0–29)**	1.7 (0–6)*	1.4 (0–42)
Mortality n(%)			
Related to abdominal trauma	1 (7.1%)*	0	0
Related to other injuries	1 (7.1%)	1 (17%)	18 (4.5%)

*ED* emergency department, *ICU* intensive care unit

*, *p* < 0.05 compared to “true negative FAST”; **, *p* < 0.01 compared to “true negative FAST”

All 14 patients with a positive FAST were hospitalised, 13 of them in the ICU and one in the surgical ward. Two patients went to the OR for explorative laparotomy (patients 4 and 9 in Table 2) and 3 patients underwent angiographic embolization (patients 8, 11 and 12). One patient died in the ED due to intra-abdominal bleeding (patient 10), and one additional patient died in-hospital from neurological complications (patient 14). All 6 patients with a false negative FAST were also hospitalised (4 in the ICU, and 2 in the surgery ward). One of them went to the OR for explorative laparotomy (patient 18) and 1 underwent angiographic embolization (patient 20). None of them died during their hospital stay. Of the 401 patients with a true negative FAST, in 49 (12%) a negative CT confirmed the findings. 285 patients were hospitalised (100 in the ICU, and 185 in the surgery ward). None of these patients went to the OR for explorative laparotomy, but one patient underwent angiographic embolization for a splenic rupture with active bleeding. A total of 18 patients with a negative FAST exam died in-hospital after presentation in the ED. Autopsy was not performed in any of these patients. However, according to the hospital charts, abdominal injuries were in none of them the presumed cause of death.

### Patient characteristics related to an adverse outcome

Univariate logistic regression analysis revealed that ISS-score, pH, base excess (BE), HCO3- concentration, aspartate transaminase (AST) concentration, activated partial thromboplastin time (APTT), and FAST-exam result were all related to an adverse outcome. Diagnostic accuracy indices for these characteristics are presented in Table 4. An elevated AST-level above 251 U/L, a BE lower than -5.7 mmol/l and an ISS score >25 all increased the likelihood of an adverse outcome significantly. However, the positive likelihood ratio of a positive FAST (34.3 [15.1–78.5]) was much higher.

**Table 4:** Diagnostic accuracy indices of patient characteristics associated with an adverse outcome in patients presenting with BAT

	Cut-off value	Sens (95% CI)	Spec (95% CI)	LR (+)	LR (-)
ISS-score	>25	78 (40–96)%	76 (72–80%)	3.3 (2.2–4.9)	0.3 (0.1–1.0)
pH	< 7.32	33 (6–76%)	80 (74–85%)	1.7 (0.5–5.4)	0.8 (0.5–1.5)
BE (mmol/l)	< −5.7	33 (6–76%)	91 (86–95)%	3.8 (1.1–12.7)	0.7 (0.4–1.3)
HCO3^-^ (mmol/l)	< 22	50 (14–86)%	53 (45–60)%	1.0 (0.5–2.4)	1.0 (0.4–2.1)
AST (U/l)	> 251	33 (9–69)%	97 (94–98)%	10.3 (3.5–30.0)	0.7 (0.4–1.1)
APTT (sec)	> 33	14 (1–58%)	95 (92–97%)	2.8 (0.4–18.7)	0.9 (0.7–1.2)
Positive FAST-result	NA	67 (31–91)%	98 (96–99%)	34.3 (15.1–78.5)	0.34 (0.13–0.86)

*LR -* negative likelihood ratio, *LR+* positive likelihood ratio, *CI* confidence interval

## Discussion

In this study, we demonstrate that the FAST exam can provide valuable prognostic information besides ISS score and clinical- and biochemical measurements in HD stable patients presenting in the ED after blunt abdominal trauma.

Previous studies have investigated the accuracy of the FAST-exam [6, 7, 19–23, 25, 26], although only a limited number of these studies were conducted in HD stable patients [19, 21]. The low sensitivity of FAST as found in our study (67%) is comparable to sensitivities reported in these studies. However, it is important to note that actual sensitivity in our study might even have been lower, since only a small amount (12%) of the negative FAST exam results in our study were confirmed by CT. When the FAST result was false negative, this remained not without consequences: 2 of the 6 patients with a false negative FAST result eventually needed a critical intervention to stabilize them (1 went to the OR for explorative laparotomy and 1 underwent an angiographic embolization). Thereby our findings stress that even in HD stable patients, one should not rely on a single negative FAST-exam to exclude serious abdominal injuries: either careful observation, or a repeated FAST-exam or additional radiological studies (preferably CT) or a combination of these should be performed.

Specificity of FAST in our study on the other hand was high (99%), which is in line with previous studies [19, 20, 22, 23]. However, when the FAST was positive additional diagnostic studies were always performed to identify the source of the bleeding and/or the extend of organ injury. Almost half of the patients with a positive FAST were treated either by exploratory laparotomy or angiographic embolization. Thereby, we can conclude that a further diagnostic work-up after an initial positive FAST-exam remains mandatory in adult patients presenting after BAT, even when they are HD stable. These patients should not be hospitalised without further diagnostic studies.

The limited sensitivity of FAST, and the fact that additional diagnostic studies are required when FAST is positive does not mean that we should abandon FAST in HD stable patients presenting after BAT. A good FAST exam takes only 30 s, and can be performed during the primary survey. Our study demonstrates clearly that, when positive, it predicts the need for a critical intervention more accurately than ISS, vital parameters or laboratory findings at presentation do. This is in line with a previous study by Deunk et al. [27] who showed that a positive FAST exam had a higher odds ratio for the prediction of the presence of injuries on CT than clinical and laboratory results in an adult population with blunt abdominal trauma. However, it should be noted that in a minority of patients in our study FAST results are false positive. These patients underwent subsequent negative CT-scanning, and were therefore exposed to radiation exposure at no clinical benefit.

Our study had several limitations. First, inherent to the retrospective design of our study, we had to cope with missing data. Although we are confident that no patients were missed during the study period (since patients were entered in the trauma registry prospectively 24/7), data on outcome/follow-up were not complete, and clinical- and biochemical data were not always available. Furthermore, since a FAST scan was performed in only a subset of the population presenting with BAT (592 out of 632 patients), selection bias might have influenced our results. With 421 patients our study population was relatively small. Only 14 patients had a positive FAST-exam, and especially for this group, missing data may have had a substantial impact on the results of logistic regression analysis.

HD stability refers to adequate blood flow and organ perfusion. However, measurement of these variables can be time-consuming. Therefore, expedient assessment of haemodynamic state must rely on simple parameters as SBP and Heart rate (HR). The chosen SBP cut-off of >90 mmHg to define HD stability in our study is fairly arbitrary, and it is debatable weather one should rely on only one parameter to define haemodynamic state. In a recent study, Hamada et al. used a combination of SBP > 90 mmHg AND HR < 110 bpm to define HD stability [28]. When we would have adopted this definition, 26 subjects would have been reclassified as HD unstable, including two subjects with a positive FAST. However, none of these subjects experienced an adverse outcome. Therefore it is questionable if this would have affected our results significantly.

FAST was not performed in 110 subjects presenting with BAT. Since it is likely that the tendency/urgency to perform a FAST is higher in subjects who are more severely injured, subjects with minor injuries might have been underrepresented in our population. Therefore, it should be stressed that our results are only applicable to populations with a similar disease severity (as reflected by ISS score), and should not be extrapolated to other populations with either a much higher-or lower ISS score.

## Conclusion

The FAST-exam can provide valuable prognostic information at minimal expenses during early stages of resuscitation in haemodynamically stable patients presenting with BAT, and should therefore not be omitted.

## Additional file

Additional file 1: Spss datafile. (SAV 134 kb)
